# A Rare Clinical Variant of Oromandibular Limb Hypogenesis Syndrome Type I B

**DOI:** 10.5005/jp-journals-10005-1338

**Published:** 2016-04-22

**Authors:** Ritesh Rambharos Kalaskar, Alkesh Godhane, Ashita Kalaskar, Swati Demble

**Affiliations:** 1 Associate Professor, Department of Pediatric Dentistry, Government Dental College and Hospital, Nagpur, Maharashtra, India; 2 Assistant Professor, Department of Pediatric Dentistry, Government Dental College and Hospital, Nagpur, Maharashtra, India; 3 Assistant Professor, Department of Oral Medicine and Radiology, VSPM Dental College and Research Centre, Nagpur, Maharashtra, India; 4 Intern, Department of Pediatric Dentistry, Government Dental College and Hospital, Nagpur, Maharashtra, India

**Keywords:** Aglossia, Environmental factors, Hypodactyli, Microstomia, OLHS, Rudimentary ears.

## Abstract

Aglossia is a rare congenital malformation that often occurs as an isolated disorder or is observed in association with other congenital deformities, particularly limb defects. We present a unique case of a 7-year-old girl with aglossia, hypodactyli, rudimentary ears, retrognathic and V-shaped mandible. Her parental history revealed intrauterine exposure of medicines. The patient had problems in difficulty in eating, speech, taste sensation and hearing. The present case does not fit into Hall’s classification of oromandibular limb hypogenesis syndrome (OLHS) which best describes hypoglossia and limb deformities. Therefore, the purpose of this article is to document the rare variant of OLHS which can be included in Hall’s classification.

**How to cite this article:** Kalaskar RR, Godhane A, Kalaskar A, Demble S. A Rare Clinical Variant of Oromandibular Limb Hypogenesis Syndrome Type I B. Int J Clin Pediatr Dent 2016;9(1):78-81.

## INTRODUCTION

Oromandibular limb hypogenesis syndrome (OLHS) (OMIM 103300) represents a spectrum of disorders affecting the tongue and limbs with an incidence of 1:175,000 live births.^1^ The etiology of this syndrome is still unknown. Both genetic and environmental factors have been proposed for the occurrence. However, intrauterine exposure of environmental factors such as radiation, medicines, nutritional deficiencies or maternal hyperthermia are the most widely accepted etiology.^2-4^ Drugs like Tigan, Benedictine, Imipramine, Diazepam, Chlorpromazine, and Meclizine are involved in the etiology, but their effect in the causation of this syndrome has not been proved.^2-4^ The patients with this syndrome often present overlapping clinical manifestations such as aglossia, hypoglossia, adactyli, and hypodactyli.^2-4^ Aglossia is an extremely rare congenital condition associated with congenital deformities such as adactyli, hypodactyli, partial anodontia, deafness, microcephaly, cleft palate, mental retardation, and hypodontia.^2^ Kettner^5^ first noted the association of aglossia with congenital deformities, whereas Rosenthal^6^ first reported the presence of aglossia and adactyli.

Hall’s classification of OLHS best describes hypoglossia and limb deformity.^7^ However, this classification fails to classify aglossia and other associated deformities. The present case describes a rare case of aglossia associated with hypodactyli, rudimentary ears and conduction deafness which can be included in Hall’s classification of OLHS.

## CASE REPORT

A 7-year-old girl reported to our department with a history of pain and swelling in the upper left back region of jaw since 3 days. The patient’s medical history was insignificant. She was the second child of a healthy nonconsanguineous parent. She has four siblings: Three sisters and one brother. Her parents and siblings are normal with no craniofacial and skeletal abnormalities. Mother gives history of hydroamenosis during her first trimester of pregnancy. Due to hydroamenosis she developed fever for which she has taken medicine from a local doctor. She was unable to remember the names of the medications. During this period she also developed rashes over her body. The child cried at birth but the obstetrician did not notice any abnormality except for the rudimentary ear. After birth she was admitted to the intensive care unit (ICU) for 10 days, thereafter she was discharged from the ICU.

**Fig. 1:**
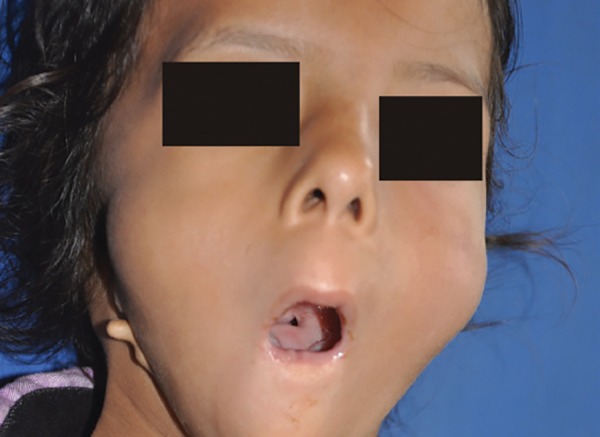
Extraoral photograph showing long narrow face, tapering chin, rudimentary ears, microstomia, and retrognathic mandible

**Fig. 2:**
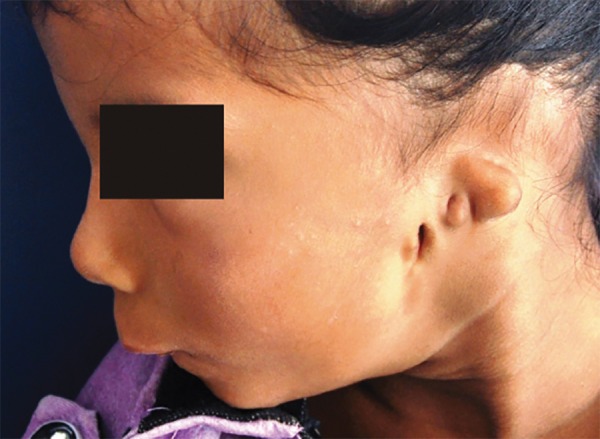
Extraoral photograph showing rudimentary ear and convex profile

**Figs 3A to C:**
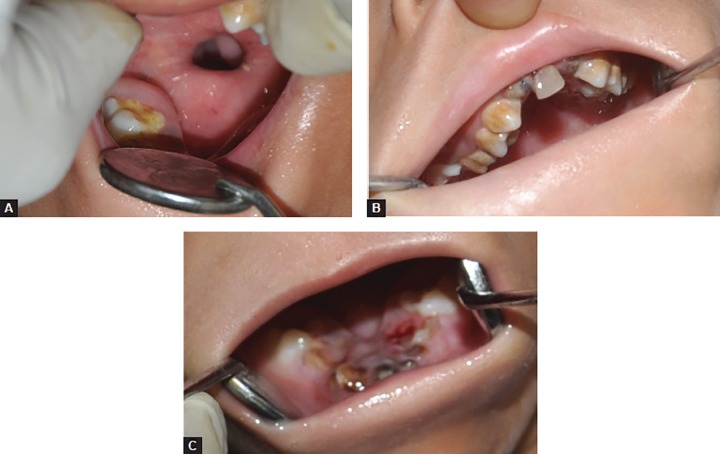
Intraoral photograph showing: (A) aglossia, (B) carious maxillary teeth, and (C) V-shaped mandible and carious teeth

General examination revealed a thin built girl with hypoplasia of left hand thumb. Extraoral examination revealed a long narrow face, tapering chin, rudimentary ears, microstomia, and retrognathic mandible (Figs 1 and 2). She had difficulty in eating, swallowing, taste sensation, speech, hearing, and breathing. She mostly had liquid diet. Lips were incompetent with convex profile (Fig. 2). Eyes, eyebrows and fingernails were normal. Weight, height and head circumference were within normal limits for her age. Her psychomotor development was not within normal parameter which was assessed using Wechsler Intelligence Scale for Children. Intraoral examination revealed aglossia, V-shaped mandible, crowding with lower anterior, constricted maxilla, palatally inclined maxillary teeth and carious teeth (maxillary and mandibular) with poor oral hygiene. The patient’s parents were unaware of aglossia until she visited our department (Fig. 3).

**Fig. 4:**
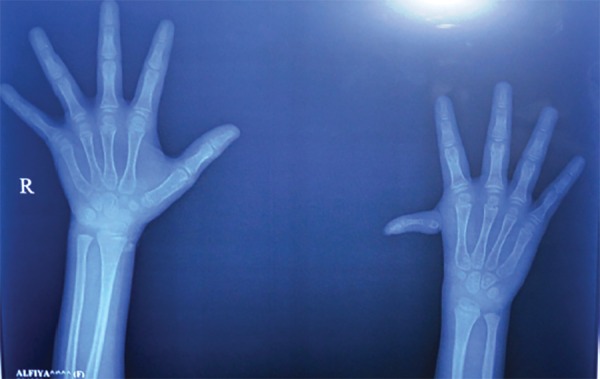
Radiograph of the hand showing hypoplastic first metacarpal of left hand with nonformation of first carpometacarpal joint

**Fig. 5:**
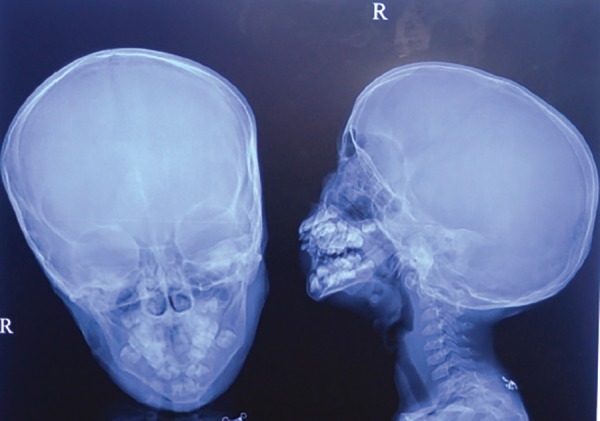
Radiograph of the skull (anteroposterior and lateral view) showing hypoplasia of the maxillary sinus, hypoplasia of the mandibular arch and V-shaped mandible

Radiograph of the hand showed hypoplastic first metacarpal of left hand with nonformation of first carpometacarpal joint (Fig. 4). Radiograph of the skull (anteroposterior and lateral view) showed hypoplasia of the maxillary sinus, hypoplasia of the mandibular arch and V-shaped mandible (Fig. 5). Panoramic radiograph was not possible as the child was very uncooperative.

Treatment plan was explained to the parents that included multiple extraction, pulpectomy and restoration. Treatment under OPD basis was not possible because of microstomia and uncooperative behavior. Patient’s parents were not ready for treatment under general anesthesia due to high-risk anesthetic complication. However, basic oral rehabilitation procedures like extraction of the root stumps and oral prophylaxis were performed under antibiotic, and analgesic coverage. Importance of excellent oral hygiene, use of daily topical fluoride, oral prophylaxis, and regular dental checkup against caries risk were explained to the patient’s parents. The patient was kept on further follow-up observation.

## DISCUSSION

Aglossia and hypoglossia are rare and sporadic congenital malformations. These malformations may occur as an isolated disorder or are observed in association with other congenital deformities, particularly limb defects.^2^ Hall, grouped these congenital malformations under OLHS and classified them into five categories with the only necessary criteria of hypoglossia and limb defect (Table 1). However, there are cases reported in the literature in which aglossia was also associated with limb and other congenital deformities.^1,8^ Preis et al^9^ noticed aglossia with hypodactyli, ventricular septal defect, anodontia, epidermoid of right eye, 6th and 7th cranial nerve palsy. In one of the recent cases Kantapura and Tanpaiboon^10^ showed association of aglossia with mental retardation, hypothyroidism, partial anodontia, microstomia and microcephaly. The present case showed association of aglossia with hypodactyli, rudimentary ear and conduction deafness. This case is the first of its kind reported in the literature. However, conduction deafness with aglossia was also reported by Higashi and Edo.^11^ Therefore, there is a need to further classify Hall’s classification based on aglossia and hypoglossia. We suggest modification for type I B Hall’s classification (Table 2).

Syndromes, such as Moebius syndrome, Hanhart syndrome, Pierre Robin syndrome and glossopalatine ankylosis syndrome should be considered in the differential diagnosis of OLHS.^9^ Moebius syndrome can be differentiated from OLHS by the presence of chest wall abnormalities and paralysis of VI and VII cranial nerve, whereas glossopalatine ankylosis syndrome has associated cleft palate with intraoral band attaching tongue, palate and maxillary alveolar ridge.^9^ Lustmann et al^2^ proposed three diagnostic characteristic features of OLHS that are hypoglossia, limb deformities, and micrognathia. However, we proposed to add aglossia in these differential diagnostic characteristic features of OLHS.

**Table 1:** **Table 1:** Hall’s classification of oromandibular limb hypogenesis syndrome

Type I		A: Hypoglossia	
		B: Aglossia	
Type II		A: Hypoglossia-hypodactylia	
		B: Hypoglossia-hypomelia	
		C: Hypoglossia-hypodactylomelia	
Type III		A: Glossopalatine ankylosis	
		B: With hypoglossia	
		C: With hypoglossia-hypodactylia	
		D: With hypoglossia-hypomelia	
		E: With hypoglossia-hypodactylomelia	
Type IV		A: Intraoral bands and fusion	
		B: With hypoglossia	
		C: With hypoglossia-hypodactylia	
		D: With hypoglossia-hypomelia	
		E: With hypoglossia-hypodactylomelia	
Type V		A: Hanhart syndrome	
		B: Charlie M syndrome	
		C: Pierre Robin syndrome	
		D: Moebius syndrome	
		E: Amniotic band syndrome	

**Table 2:** **Table 2:** Modification of type I B Hall’s classification

*Hall‘s* *classification*		*Modified* *subtypes*		*Clinical Features*		*References*	
Type I B		Type I		Isolated aglossia		Salles F et al,	
(Aglossia)		B.1				Gupta S	
		Type I		Aglossia with		Nevin NC et al^1^	
		B.2		adactyli		Purohit et al^8^	
		Type I		Aglossia with		Preis et al,^9^	
		B.3		hypodactyli (mental retardation, cardiac defect, anodontia, hypothyroidism)		Kantapura and Tanpaiboon^10^	
		Type I		Aglossia with		Higashi and	
		B.4		rudimentary ear (deafness)		Edo,^11^ present case	

Literature acknowledges the interaction of both genetic and environmental factors in the OLHS etiology. Almost all cases reported in the literature are sporadic but few have intrafamilial history. Till date, no genetic mutation or chromosomal abnormalities have been identified for this syndrome.^2,3^ However, the proposed etiology is heredity, maternal hyperthermia and positive drug history during pregnancy. The present case gave a positive prenatal history of maternal fever and drug during pregnancy. Evidence of maternal hyperthermia causing OHLS exists.^12,13^ Maternal fever at/above 102°F between 4 and 14 weeks of pregnancy results in a range of defects including limb reduction, central nervous system defects, facial dysmorphogenesis and fetal death. The nature of anomalies is related to the extent, duration and timing of the maternal fever. Based on this, there is a need to explore maternal hyperthermia and drug during pregnancy as a cause of the syndrome.

Tongue plays an important role in sucking, speech, mastication, swallowing, taste perception, development of jaws and occlusion. Though tongue is deficient or absent, in majority of the cases, activities like swallowing and speech improve with time.^12-14^ This was possible due to adaptive mechanism of the orofacial structure that assists in feeding and improves speech, swallowing and taste sensation.^12-14^

Timely preventive protocol, such as oral prophylaxis, pit and fissure sealants, topical fluoride application should be instituted for maintenance. Functional and esthetic rehabilitation of patient with aglossia is difficult and requires a multidisciplinary and multistep approach. Correction of malocclusion and jaw anomalies would require orthodontic and surgical treatment.^15^ Therefore, early diagnosis, sustained care and multidisciplinary approach are recommended for the management of such patients.

## References

[1] Nevin NC, Burrows D, Allen G, Kernoham DC (1975). Aglossia adactyli syndrome. J Med Genet.

[2] Lustmann J, Lurie R, Struthers P, Garwood A (1981). Hypoglossia-hypodactylia syndrome: report of 2 cases. Oral Surg Oral Med Oral Pathol.

[3] Meundi MA, Nair GR, Sreenivasan P, Raj AC (2013). Oromandibular limb hypogenesis syndrome type II B: case report of hypoglossia-hypodactyly. Case Rep Dent.

[4] Firth HV, Boyd PA, Chamberlain PF, Mackenzie IZ, Morriss GM, Huson SM (1994). Analysis of limb reduction defect in babies exposed to chorionic villus sampling. Lancet.

[5] Kettner MR (1907). Kongenitaler Zungendefekt. Deutsch Med Wehnschr.

[6] Rosenthal R (1932). Aglossia congenita. A report of case of the condition combined with other congenital malformations. Am J Dis Child.

[7] Hall BD (1971). Aglossia-adactylia. Birth defects Orig Artic Ser.

[8] Purohit SK, Kunta SM, Rao PP, Thatte RL (1989). An interesting case of aglossia-adactyli syndrome. Br J Plast Surg.

[9] Preis S, Majewski F, Hantschman R, Schumacher H, Lenard HG (1996). Goldenhar, Mobius and hypoglossal-hypodactyli anomalies in a patient; syndrome or association?. Eur J Pediatr.

[10] Kantapura P, Tanpaiboon P (2003). Thyroid dysfunction in a patient with aglossia. Am J Med Genet A.

[11] Higashi K, Edo M (1996). Conductive deafness in aglossia. J Laryngol Otol.

[12] Graham JM Jr, Edwards MJ (1998). Teratogen update: gestational effects of maternal hyperthermia due to febrile illness and resultant pattern of defects in humans. Teratology.

[13] Wadhwani P, Mohammad S, Sahu R (2007). Oromandibular limb hypogenesis syndrome, type IIA, hypoglossia-hypodactylia: a case report. J Oral Pathol Med.

[14] Gupta S (2012). Isolated aglossia congenital: a rare case of oroman-dibular limb hypogenesis syndrome type I B. J Oral Maxillofac Pathol.

[15] Weingarten RT, Walner DL, Holinger LD (1993). Tongue hypoplasia in newborn. Int J Pediatr Otorhinolaryngol.

